# Dihydroartemisinin Promoted Bone Marrow Mesenchymal Stem Cell Homing and Suppressed Inflammation and Oxidative Stress against Prostate Injury in Chronic Bacterial Prostatitis Mice Model

**DOI:** 10.1155/2021/1829736

**Published:** 2021-12-15

**Authors:** Shen Li, Yongzhang Li, Xiaozhe Su, Aiyun Han, Yang Cui, Shuyue Lv, Jin Zhang, Chao Li

**Affiliations:** ^1^Department of Urology, Shijiazhuang City People's Hospital, Shijiazhuang 050000, Hebei, China; ^2^Department of Urology, Hebei Province of Chinese Medicine, Shijiazhuang 050011, Hebei, China

## Abstract

Although bone marrow mesenchymal stem cells (BMMSCs) are effective in treating chronic bacterial prostatitis (CBP), the homing of BMMSCs seems to require ultrasound induction. Dihydroartemisinin (DHA) is an important derivative of artemisinin (ART) and has been previously reported to alleviate inflammation and autoimmune diseases. But the effect of DHA on chronic prostatitis (CP) is still unclear. This study aims to clarify the efficacy and mechanism of DHA in the treatment of CBP and its effect on the accumulation of BMMSCs. The experimental CBP was produced in C57BL/6 male mice via intraurethrally administered *E. coli* solution. Results showed that DHA treatment concentration-dependently promoted the accumulation of BMMSCs in prostate tissue of CBP mice. In addition, DHA and BMMSCs cotreatment significantly alleviated inflammation and improved prostate damage by decreasing the expression of proinflammatory factors such as TNF-*α*, IL-1*β*, and chemokines CXCL2, CXCL9, CXCL10, and CXCL11 in prostate tissue of CBP mice. Moreover, DHA and BMMSCs cotreatment displayed antioxidation property by increasing the production of glutathione peroxidase (GSH-Px), SOD, and decreasing malondialdehyde (MDA) expression. Mechanically, DHA and BMMSCs cotreatment significantly inhibited the expression of TGF*β*-RI, TGF*β*-RII, phosphor (p)-Smad2/3, and Smad4 in a dose-dependent manner while stimulated Smad7 expression in the same manner. In conclusion, our findings provided evidence that DHA effectively eliminated inflammatory and oxidative stress against prostate injury, and this effect involved the TGF-*β*/Smad signaling pathway in CBP.

## 1. Introduction

Chronic prostatitis (CP) is a common disease in the urology clinic. The clinical manifestations of CP include abnormal urination, pain, and sexual dysfunction, especially symptoms of voiding dysfunction [1]. Based on symptomatology, and the presence or absence of leukocytes in semen and prostatic fluid, CP was classified into three categories, namely, chronic bacterial prostatitis (CBP), chronic nonbacterial prostatitis (CNP), and experimental autoimmune prostatitis (EAP) [1]. The traditional treatment of CP was mainly antibiotics, *α*-blockers, and anti-inflammatory drugs, combined with psychological and physical therapies [2]. However, the treatment effects of these methods are not very satisfactory. Due to the multitargeted therapy and fewer side effects of traditional Chinese medicines (TCMs), there are more and more research studies that have focused on the prevention and treatment of CP with TCM [3]. Many TCM compound preparations and their active components such as *Abacopteris penangiana* [4], Bazhengsan [5], and flavonoids [6] were effective in the treatment of CP.

Artemisinin (ART) was a well-known clinically important antimalarial drug, which was extracted from Artemisia annual leaves and was first discovered and reported by Professor Tu Youyou, an outstanding pharmacologist in China [7]. Dihydroartemisinin (DHA, C_15_H_24_O_5_) is a sesquiterpenoid compound and is an important derivative of ART. As DHA research progresses, the pharmacological benefits of DHA were not only limited to the treatment of malaria but also showed great promise in anti-inflammatory and immune regulation. Previous studies have found that DHA was effective in the treatment of inflammatory diseases and oxidative stress, especially for acute kidney injury [8], pulmonary fibrosis [9], and airway inflammation [10]. It is preliminarily confirmed that DHA could inhibit inflammatory bowel disease (IBD) by regulating the balance of Th17/Treg cells [11]. Furthermore, more and more evidence showed that DHA also had powerful neuroprotective [12], antitumor [13, 14], and inhibiting angiogenic effects [15]. DHA was widely reported inhibiting the progression of prostate cancer [16, 17]. The occurrence of prostate cancer and prostatitis was closely related [18]. However, the function and potential molecular mechanism of DHA on prostatitis are unclear.

Bone marrow mesenchymal stem cells (BMMSCs) are a stem cell population with rapid population proliferation, multidirectional differentiation, low immunogenicity, immune suppression, and tissue repair ability [19]. BMMSCs have been applied in clinical inflammatory disease treatment. Intriguingly, the previous research in CBP rats found that microbubble-mediated ultrasound-induced BMMSC accumulation inhibited inflammation and decreased TNF-*α* and IL-1*β* expressions [20]. In this study, we aimed to investigate the role of DHA treatment and its involvement in BMMSC homing in a mouse model of CBP.

## 2. Methods and Materials

### 2.1. Reagents

DHA was purchased from KPC Pharmaceuticals, Inc. (Chongqing, China). Product batch number: C00220160402.

### 2.2. Animals

C57BL/6 male mice (SPF grade, 9–11 weeks) were purchased from Dashuo Animal Experiment Co., Ltd. (Chengdu, Sichuan; license number: SCXY (Chuan) 2020–034). The feeding environment was 25 ± 1 °C, relative humidity 50%–60%, and light/darkness for 12-h circulation. The mice are allowed to freely eat and drink.

### 2.3. Animal Grouping and Treatment

The experimental protocol for the care and use of laboratory animals was approved by the Experimental Animal Ethics Committee of Shijiazhuang City People's Hospital (2021097). The mice were randomly divided into 5 groups (*n* = 6), namely, the control group, model group, BMMSCs group, low-dose DHA + BMMSCs group, and high-dose DHA + BMMSCs group. All mice were anesthetized with 1% sodium pentobarbital (50 mg/kg, Merck, Germany) prior to treatment. For the control group, the male mice were instilled with 200 *μ*l phosphate buffer saline (PBS); for the model group, the mice were intraurethrally administered with 200 *μ*l of *E. coli* solution (2 × 10^6^ cfu/ml). After modeling, all mice were normally fed for 4 weeks. Then, for the BMMSC group, the mice were injected with 30 *μ*l of PBS buffer containing 1 × 10^7^ GFP-BMMSCs in the tail vein; for DHA low- and high-dose groups, the mice were given DHA 10 mg/kg/d and 20 mg/kg/d by gavage, respectively. The control and model groups were given 0.9 g/L saline 3 ml/d by gavage. All mice were raised in an SPF environment for another 2 weeks. Finally, the mice were anesthetized with 1% sodium pentobarbital (50 mg/kg) and then euthanized. The prostate tissue was removed for subsequent analysis.

### 2.4. Cell Culture

Mouse BMMSCs were purchased from Procell (#CP-M131, Wuhan, China). The cells were maintained in DMEM/F-12 medium supplemented with 10% fetal bovine prostate tissue (FBS; Gibco, Grand Island, NY) at 37°C with 5% CO_2_ in a humidified incubator. Then, BMMSCs (3 × 10^3^/well) were seeded in a 96-well plate and cultured in an incubator (37°C, 5% CO_2_) for 24 h. Subsequently, 100 *μ*l diluent of GFP-LUC adenovirus (1 × 10^9^ cfu/ml) was transfected into BMMSCs and cultured for 24 h. A fluorescence microscope was used to observe the expression of fluorescence and determine the transfection efficiency.

### 2.5. CCK-8 Assay

The cell viability of GFP-BMMSCs was measured using the Cell Counting Kit-8 (CCK-8, Thermo Fisher Scientific) according to the instructions of the manufacturer. Absorbance was recorded at 450 nm.

### 2.6. Hematoxylin and Eosin (H&E) Stain

Mice prostate tissues were fixed in 4% paraformaldehyde for 24 h and embedded in paraffin to a histopathological study by H&E stain. In brief, each mice prostate paraffin section was deparaffinized and dehydrated with xylene and graded ethanol. After staining with hematoxylin and eosin, the paraffin sections were washed with distilled water and dehydrated with graded ethanol and xylene. The inflammation degree of each prostate tissue was evaluated using a light microscope under 5 random fields.

### 2.7. Western Blot Analysis

Prostate tissues were homogenized using a homogenizer with magnetic beads. Total protein was manufactured using RIPA buffer (Cell Signaling Technology, Inc.). The concentration of protein was determined by a BCA kit (Sigma-Aldrich; Merck KGaA, Germany). Total protein (30 *μ*g/sample) was separated via 10% SDS-PAGE. And then, the separated proteins were transferred to nitrocellulose membranes. The membranes were blocked with 5% nonfat dried milk overnight at 4°C and incubated with the following corresponding protein antibodies: TGF*β*-RI (1 : 1000, Abcam, #31013), TGF*β*-RII (1 : 1000, Abcam, #186838), phosphor (p)-Smad2/3 (1 : 800, Abcam, #254407), Smad4 (1 : 1000, CST, #38454), Smad7 (1 : 1000, Santa Cruz, #365846), and *β*-actin (1/1000, Boster, #BM0627). Then, the membranes were washed with Tris-buffered saline/0.1% Tween (TBST) and incubated for 1.5 hours with HRP goat anti-rabbit IgG (1 : 2000, Abcam, #6721). The bands were visualized using the ECL system (Affinity Biosciences, Cincinnati, Ohio, USA), and *β*-actin was used as an internal control. The net optical density was measured using Quantity One software (Bio-Rad).

### 2.8. Immunofluorescence (IF) Stain

IF stain was used to determine the expression of CD29, CD44, and CD45 in mouse prostate tissue. Mouse prostate paraffin sections (8 *μ*m) were fixed in acetone for 10 min and rinsed 3 times with PBS. Then, the tissue sections were, respectively, incubated at 4°C overnight with rabbit anti-mouse CD29, CD44, and CD45 monoclonal antibodies (1 : 100, Bioss Biotechnology Ltd., Beijing, China). Sections were rinsed with PBS and incubated with bovine anti-rabbit IgG-FITC (Santa Cruz, USA) for 30 min at room temperature. Finally, sections were incubated with DAPI and observed with a fluorescent microscope.

### 2.9. Enzyme-Linked Immunosorbent Assay (ELISA)

The contents of TNF-*α*, IL-1*β*, CXCL2, CXCL9, CXCL10, CXCL11, MDA, SOD, and GSH-Px in prostate tissue of CBP mice were measured with ELISA kits (Takara, Tokyo, Japan) following the instructions of the manufacturer. The absorbance measured at 450 nm wavelength was estimated using an enzyme-linked immune monitor (Thermo Fisher Scientific, Inc., USA). The concentrations of TNF-*α*, IL-1*β*, CXCL2, CXCL9, CXCL10, CXCL11, MDA, SOD, and GSH-Px in the sample were calculated from the standard curve.

### 2.10. Statistical Analysis

The data were represented as mean ± standard deviation. Statistical analysis was performed using SPSS 20.0 (IBM Corp.). One-way analysis of variance (ANOVA) with Tukey's post hoc test of means was used for multiple group comparisons. For comparisons between two groups, Student's unpaired *t*-test was used to assess statistical significance. Differences with a *P* < 0.05 were considered to indicate statistical significance.

## 3. Results

### 3.1. Observation of GFP-LUC Adenovirus-Transfected BMSC Viability

**Figure 1:**
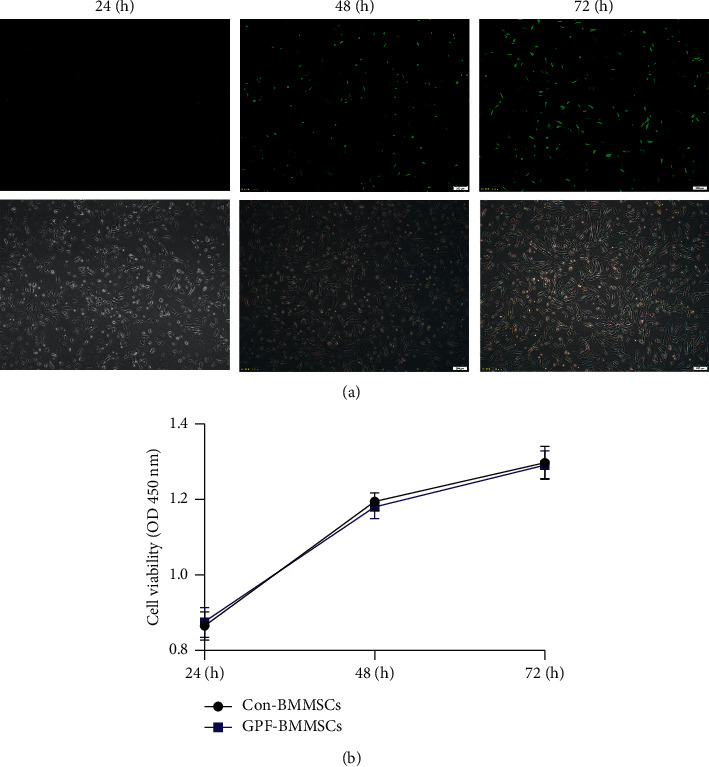
Observation of GFP-LUC adenovirus-transfected BMSC viability. (a) Green fluorescent levels in BMMSCs at 24 h 48 h and 72 h after transfection with GFP-LUC adenovirus (×100 magnification). The scale bar indicates 200 *μ*m. (b) Cell viability was tested using the CCK-8 assay. For B mean ± standard errors of means are reported.

As shown in Figure 1, the green fluorescence was observed in BMMSCs after 48 h and 72 h of GFP-LUC adenovirus transfection, which did not affect the viability of BMMSCs. Thus, the GFP-labeled BMMSCs were then injected into the tail vein for the treatment of CBP mice.

### 3.2. DHA Promoted Homing of BMMSCs to Prostate Tissue

**Figure 2:**
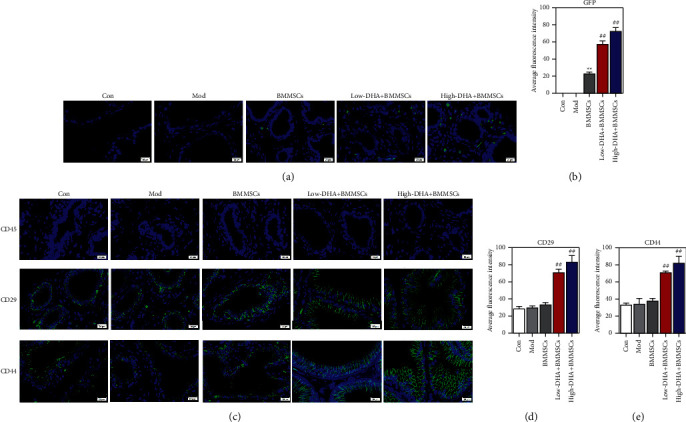
DHA promoted homing of BMMSCs to prostate tissue. (a) Fluorescence images showing GFP + cells in prostate tissue (×200 magnification). (b) Average fluorescence intensity of GFP. (c-e) Fluorescence images and average fluorescence intensity of CD45, CD29, and CD44 levels in prostate tissue (×200 magnification). The scale bar indicates 20 *μ*m. ^*∗∗*^denotes *P* < 0.01 vs con group and ##denotes *P* < 0.01 vs BMMSCs group. For B, D, and E, mean ± standard errors of means are reported.

As shown in Figures 2(a) and 2(b), compared with the control group and BMMSC alone injection group, DHA dose-dependently promoted the aggregation of BMMSCs into prostate tissue, as evidenced by increased levels of green fluorescence in prostate tissue. MSCs were able to positively express CD29 and CD44 whereas negatively for CD45. Our IF stain results showed that CD29 and CD44 protein levels were higher in prostate tissue of DHA administration mice than in the control group and BMMSC alone injection group (Figures 2(c)–2(e)). However, there was no CD45 expression in each group (Figure 2(c)). These data reflected that DHA obviously promoted homing of BMMSCs to prostate tissue in CBP mice.

### 3.3. Effect of DHA and BMMSC Cotreatment on Histopathological Features and Inflammatory Mediators in CBP Mice

**Figure 3:**
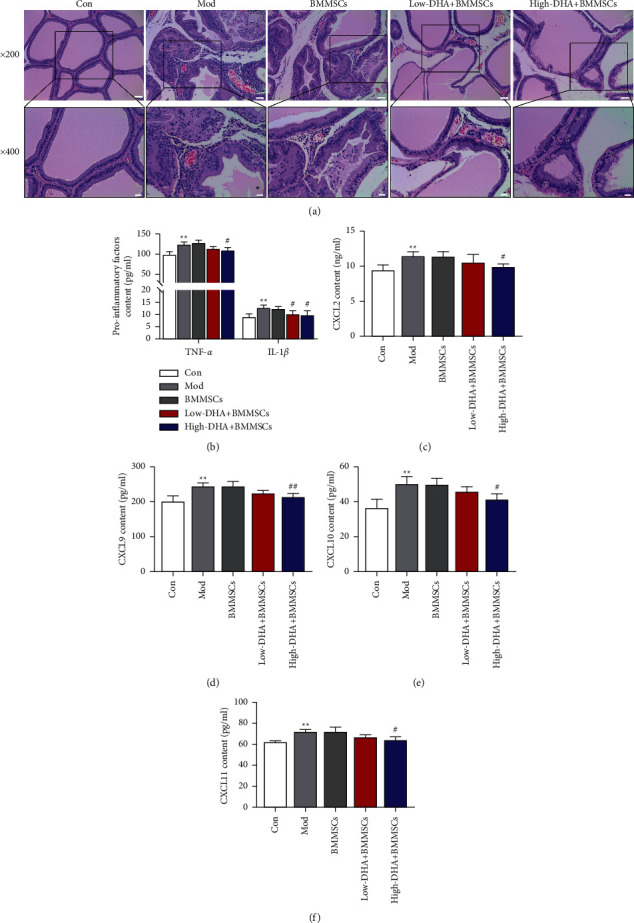
Effect of DHA and BMMSC cotreatment on histopathological features and inflammatory mediators in CBP mice. (a) H&E stain of prostate tissue from C57BL/6 mice in the different groups. The scale bar indicates 50 *μ*m (×200 magnification) and 20 *μ*m (×400 magnification). (b–f) Quantifications of TNF-*α*, IL-1*β*, CXCL2, CXCL9, CXCL10, and CXCL11 levels in prostate tissue of all groups were examined using an ELISA assay. ^∗∗^denotes *P* < 0.01 vs con group, # denotes *P* < 0.05 vs BMMSCs group, and ## denotes *P* < 0.01 vs BMMSCs group. For (b)–(f), mean ± standard errors of means are reported.

Next, we analyzed the pathological injury of mice prostate tissue. Histological analysis showed that in the control group, the glandular cavity epithelial cell layer had a complete structure and was arranged in a columnar shape (Figure 3(a)). There was no fibrous tissue hyperplasia and infiltrating inflammatory cells in the interstitium (Figure 3(a)). Both the CBP group and the CBP + BMMSC group showed the necrosis of epithelial cells. A large number of lymphocytes and plasma cells were infiltrated in the interstitium (Figure 3(a)). Low-dose and high-dose DHA significantly improved the pathological damage of prostate tissue in a concentration-dependent fashion (Figure 3(a)). Furthermore, ELISA analysis revealed upregulation of TNF-*α* and IL-1*β* in prostate tissue of CBP mice, which was inhibited by cotreatment of DHA and BMMSCs (Figure 3(b)).

The chemokines of the CXC family were reported to produce obvious proinflammatory effects in a series of diseases, especially participated in the formation and development of benign prostate hyperplasia (BPH) and prostate cancer [21]. Thus, we also detected the expression levels of chemokines in prostate tissue of CBP mice by ELISA assay. The secretions of CXCL2, CXCL9, CXCL10, and CXCL11 chemokines were increased in the model group and BMMSC alone treatment group, which were blocked by low/high-dose DHA and BMMSC cotreatment (Figures 3(c)–3(f)).

### 3.4. DHA and BMMSCs Cotreatment Attenuated Oxidative Stress in CBP Mice

**Figure 4:**
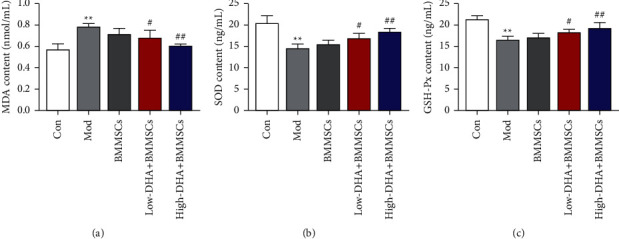
DHA and BMMSC cotreatment attenuated the oxidative stress in CBP mice. Expression of oxidative stress-related factors MDA (a), SOD (b), and GSH-Px (c) in the prostatic tissues of mice was detected by ELISA. ^∗∗^denotes *P* < 0.01 vs con group, # denotes *P* < 0.05 vs BMMSCs group, and ## denotes *P* < 0.01 vs BMMSCs group. For all the graphs, mean ± standard errors of means are reported.

Several clinical studies demonstrated oxidative stress played an essential role in the progression of CP [22–24]. To confirm that the cotreatment of DHA and BMMSCs can also improve oxidative stress in CBP mice, we tested MDA, SOD, and GSH-Px levels in prostate tissue of CBP mice using ELISA kits. A statistically significant increase in the model group was documented in terms of MDA activity (Figure 4(a)). Decreased SOD and GSH-Px activities were also found in the model group and BMMSC alone treatment group (Figures 4(b) and 4(c)). Furthermore, compared with those in the BMMSC alone treatment group, the SOD and GSH-Px activities were increased in the low-dose DHA + BMMSC group and high-dose DHA + BMMSC group, while the MDA level was decreased (Figures 4(a) and 3(c)).

### 3.5. DHA and BMMSC Cotreatment Inhibited TGF-*β*/Smad Signaling Pathway in CBP Mice

**Figure 5:**
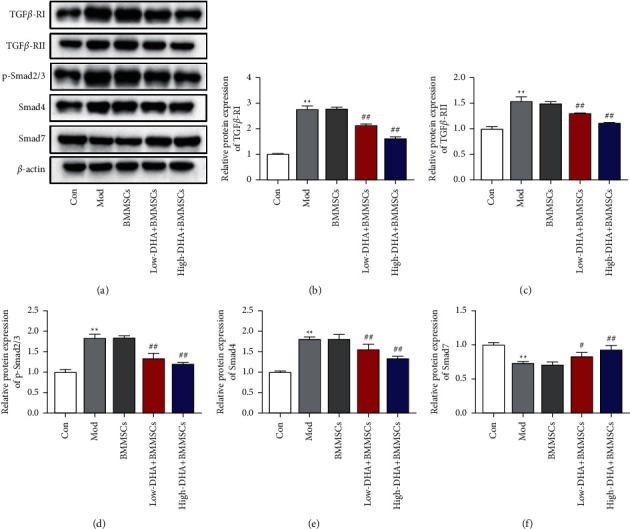
DHA inhibited TGF-*β*/Smad signaling pathway in CBP mice. (a) Representative bands of Western blot analysis. Expression of TGF*β*-RI (b), TGF*β*-RII (c), p-Smad2/3 (d), Smad4 (e), and Smad7 (f) in the prostatic tissues of mice was detected by Western blot. ^∗∗^denotes *P* < 0.01 vs con group, # denotes *P* < 0.05 vs BMMSCs group, and ## denotes *P* < 0.01 vs BMMSCs group. For (b)–(f), mean ± standard errors of means are reported.

Finally, we preliminarily investigated the mechanism of improvement of DHA in mice with prostatitis. The results indicated that in prostate tissue of CBP mice and BMMSC alone-treated mice, the expression of TGF*β*-RI, TGF*β*-RII, p-Smad2/3, and Smad4 was markedly increased (Figures 5(a)–5(e)). After low-dose and high-dose DHA cotreatment with BMMSCs, all elevated levels of these core proteins in the TGF-*β*/Smad signaling pathway were reversed in prostate tissue (Figures 5(a)–5(e)). In addition, DHA and BMMSC cotreatment dose-dependently suppressed the downregulation of Smad7 induced by prostatitis (Figures 5(a) and 5(f)).

## 4. Discussion

In this study, the results of significant accumulation of BMMSCs observed in the prostate tissue of the DHA group confirmed a possible link between DHA treatment and BMMSC homing. Furthermore, our results evaluated the effect of DHA and BMMSC cotreatment on oxidative stress and inflammation in CBP mice.

Earlier studies showed that mesenchymal stem cells (MSCs) played the role of inhibiting the inflammatory response through inhibiting T cell, B cell, and antigens that presented cell proliferation and suppressing proinflammatory factor generation [25]. MSC concentration-dependently inhibited the secretion of TNF-*α* and IL-12 from dendritic cells and promoted anti-inflammatory factor IL-10 expression [26]. Meanwhile, MSCs induced division arrest T cells in G0/Gl phase and inhibited Th1-cell proliferation [27]. In addition, TNF-*α* and IL-12 stimulated MSCs to secrete T-cell chemokines such as CXCL9, CXCL10, and CXCL11, which further promoted T-cell aggregation toward MSCs, thereby fully exerting immunoregulatory capacity [28]. Moreover, BMSCs had excellent treatment effects on inflammatory disease. In a rat model of bronchial asthma, intravenously injected BMSCs specifically accumulated in the lung inflammation after 1 hour. Subsequently, BMSCs reduced the levels of Th2 cell-related inflammatory factor levels in bronchial lavage fluid and IgGl and IgE levels in serum, thereby reducing the inflammatory response [29]. In the treatment of *E. coli* pneumonia, MSCs could reduce bacterial growth in the bronchoalveolar lavage (BAL) fluid and pulmonary inflammatory response by secreting antibacterial peptide hCAP-18/LL-37 [30]. However, our results indicated that BMMSC alone treatment did not seem to exert a favorable anti-inflammatory effect in the CBP model. This may be due to the blood-prostate barrier preventing the efficient accumulation of BMMSCs into the prostate tissue [31]. Importantly, in our CBP model, we discovered that DHA treatment promoted the accumulation of BMMSCs into the prostate tissue in a concentration-dependent manner. Hence, we deduced that DHA treatment, accompanied by BMMSCs homing, was likely to play a role in improving CBP injury.

We then investigated the effect of cotreatment with DHA and BMMSCs on inflammation in CBP mice. This study demonstrated that high-dose DHA and BMMSC cotreatment markedly diminished inflammation in prostate tissue of CBP mice, which was manifested as decreases in the expression levels of proinflammatory factor TNF-*α*, IL-1*β*, and chemokines CXCL2, CXCL9, CXCL10, and CXCL11. TNF-*α* is an important proinflammatory cytokine secreted by mononuclear macrophages, neutrophils, and T cells [32]. IL-1*β* promoted the release of protein hydrolases from neutrophils and activated T cells to accumulate in the prostate tissue, finally causing an immune response [33]. The study found that TNF-*α* and IL-1*β* levels were significantly higher in prostatic secretions of patients with CP [34]. Moreover, increased levels of chemokines have been described in clinical samples from chronic prostatitis/chronic pelvic pain syndrome (CP/CPPS) patients [35]. DHA was previously reported to hinder the production of proinflammatory cytokines IL-1*β*, TNF-*α*, and IL-6 via regulating NF-*κ*B signaling in acute lung injury (ALI) [36]. Moreover, DHA also exerted an anti-inflammatory effect through the deactivation of NLRP3 inflammasome and p38 MAPK signaling [37]. DC32, a dihydroartemisinin derivative, was found to significantly inhibit the transcription of chemokines (CXCL12 and CX3CL1) and IL-6 in rheumatoid arthritis (RA) [38]. These studies were consistent with our results showing the excellent anti-inflammatory effect of DHA and BMMSC cotreatment.

Studies have shown that the development of CP was also closely related to oxidative stress [39, 40]. CBP patients produced excessive amounts of ROS in plasma that disrupted the intracellular environment and formed toxic products such as lipid peroxide MDA [24]. SOD participated in the metabolism of reactive oxygen species, and GSH-Px inhibited the accumulation of lipid peroxidation, both of which protected cells from oxidative damage. It was found that in CP/CPPS model rats, lycopene treatment upregulated GSH-Px and SOD levels and downregulated MDA content [41]. In this study, as observed through ELISA assays, DHA and BMMSC cotreatment also significantly eliminated oxidative stress in CBP mice that suppressed the production of MDA level and promoted the levels of SOD and GSH-Px in prostate tissue. Therefore, SOD, MDA, and GSH-PX indirectly reflect the oxidation resistance of DHA and BMMSC cotreatment in CBP. Similarly, previous studies explained that DHA treatment significantly decreased oxidative damage to attenuate lung injury [42] and kidney injury [8].

Previous reports have suggested that TGF-*β*/Smad signaling played critical roles during inflammation and oxidative stress [43, 44]. Of the signaling cascade, the heterotrimer formed by TGF-*β*, TGF*β*-RI, and TGF*β*-RII phosphorylated Smad2/3 proteins and ultimately initiated transcription of the relevant target gene with the cooperation of Smad4 [45]. On the other hand, the combination of Smad7 and TGF-*β*RI could hinder the activation of Smad2/3 and control TGF-*β* signaling in a negative feedback manner [45]. TGF-*β*/Smad signaling pathway has been long considered as a key mediator in the suppression of inflammation, such as in renal inflammation [46] and rheumatoid arthritis [47]. However, pinocembrin-inhibited TGF-*β*/Smad signaling ameliorated oxidative stress and inflammation in a liver fibrosis model [44]. We found that DHA and BMMSC cotreatment inhibited the activation of the TGF-*β*/Smad signaling pathway in prostate tissue of CBP mice. These opposite results may be due to the interaction of TGF-*β*/Smad with other cytokine signaling in the inhibition and promotion of inflammation [43].

In conclusion, DHA performed potent anti-inflammatory and antioxidant effects in CBP mice by promoting the accumulation of BMMSCs into the prostate tissue. Meanwhile, the homing of BMMSCs promoted by DHA inhibited the activation TGF-*β*1/Smad signaling pathway. The combination therapy of DHA and BMMSCs has shown excellent promise in the treatment of CP.

## Data Availability

The datasets used or analyzed during the current study are available from the corresponding author on reasonable request.
